# Introducing the Special Issue Honoring Lihong V. Wang, Pioneer in Biomedical Optics

**DOI:** 10.1117/1.JBO.29.S1.S11500

**Published:** 2024-06-06

**Authors:** Xueding Wang, Mark Anastasio, Hao Zhang, Sava Sakadzic, Song Hu, Liang Gao

**Affiliations:** a University of Michigan, School of Medicine, Ann Arbor, Michigan, United States; b University of Illinois Urbana - Champaign, The Grainger College of Engineering, Department of Bioengineering, Urbana, Illinois, United States; c Northwestern University, Department of Biomedical Engineering, Evanston, Illinois, United States; d Harvard Medical School, Department of Radiology, Boston, Massachusetts, United States; e Athinoula A. Martinos Center for Biomedical Imaging, Mass General Brigham, Charlestown, Massachusetts, United States; f Washington University in St. Louis, Department of Biomedical Engineering, St. Louis, Missouri, United States; g University of California Los Angeles, Department of Bioengineering, Los Angeles, California, United States

## Abstract

The editorial concludes the JBO Special Issue Honoring Lihong V. Wang, outlining Prof. Wang’s salient contributions to advancing the field of biomedical optics.

We present this special issue of the *Journal of Biomedical Optics* (JBO) in honor of Prof. Lihong Wang, a leading pioneer in the field of biomedical optics. Prof. Wang is the Bren Professor of Medical Engineering and Electrical Engineering and currently serves as the Executive Officer of the Andrew and Peggy Cherng Department of Medical Engineering at the California Institute of Technology. Prof. Wang is renowned for his many seminal contributions that have broadly impacted the field of biomedical optics. For example, his laboratory was the first to report functional photoacoustic tomography, 3D photoacoustic microscopy (PAM), photoacoustic endoscopy, photoacoustic reporter gene imaging, the photoacoustic Doppler effect, the universal back-projection algorithm for photoacoustic tomography image reconstruction, microwave-induced thermoacoustic tomography, ultrasound-modulated optical tomography, time-reversed ultrasonically encoded (TRUE) optical focusing, nonlinear photoacoustic wavefront shaping (PAWS), compressed ultrafast photography, Mueller-matrix optical coherence tomography, and optical coherence computed tomography. On these and other topics, Prof. Wang has published over 600 journal articles to date, which have been cited over 106,000 times. According to the recent “list of world’s top-cited scientists” managed by Stanford and Elsevier, Dr. Wang is ranked #1 in the subfield of “Optics” and #4 in the subfield of “Medical Imaging” (2023). Prof. Wang’s book *Biomedical Optics: Principles and Imaging* was one of the first in the field and received the Joseph W. Goodman Book Writing Award. He was also the editor-in-chief of JBO from 2010 to 2017.

Prof. Wang’s research excellence has been universally recognized. He is a member of the National Academy of Engineering and a fellow of the National Academy of Inventors, American Association for the Advancement of Science, and the International Academy of Medical and Biological Engineering. Additionally, he is a fellow of SPIE, IEEE, Optica, and the Electromagnetics Academy. His professional awards are too numerous to list, and include the Michael S. Feld Biophotonics Award, the Britton Chance Biomedical Optics Award, the IEEE Biomedical Engineering Award, the IEEE Technical Achievement Award, and the C.E.K. Mees Medal. His distinctive awards from the National Institutes of Health (NIH) include the NIH Director’s Transformative Research Award, NIH Director’s Pioneer (DP1) Award, and NIH/NCI Outstanding Investigator Award. He also received an honorary doctorate degree from Lund University (Sweden) for his contributions to the field of biomedical imaging.

To honor Prof. Wang’s achievements, this special issue is dedicated to his contributions to biomedical optics. A brief summary of his specific contributions to biomedical photoacoustic imaging, Monte Carlo modeling of photon transport in biological tissues, compressed ultrafast photography, and time reversal and wavefront shaping in scattering media are surveyed below and have provided a foundation for the papers reported in the special issue.

## 1 Biomedical Photoacoustic Imaging

**Fig. 1:**
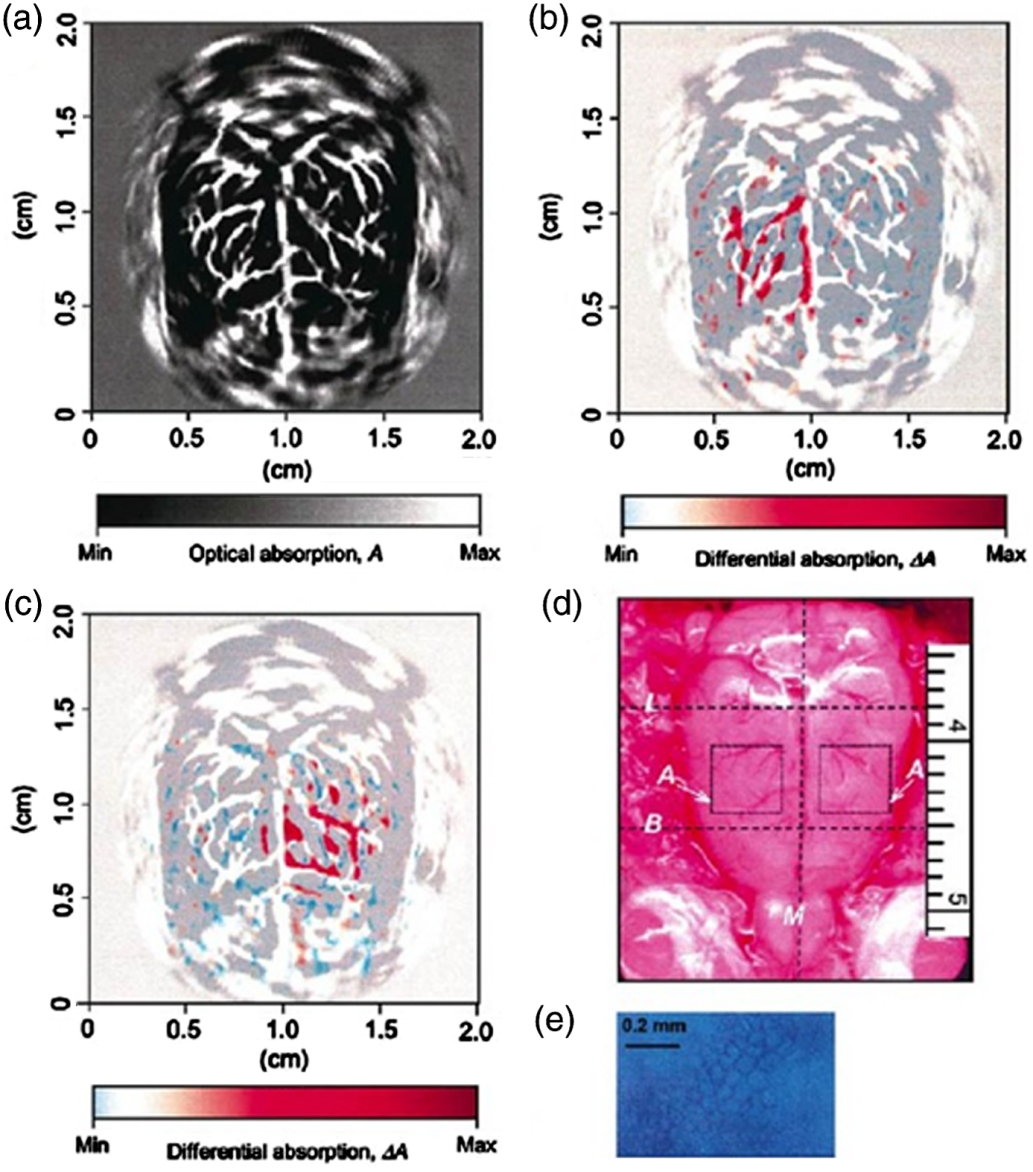
**Functional photoacoustic computed tomography (PACT).** (a) Noninvasive PACT image of the vascular pattern in the superficial layer of the rat cortex acquired with the skin and skull intact. The matrix size of the image was 1,000 (horizontal) × 1,000 (vertical), showing a 2.0 cm × 2.0 cm region. (b), (c) Noninvasive functional PACT images corresponding to left-side and right-side whisker stimulation, respectively, acquired with the skin and skull intact. These two maps of functional representations of whiskers are superimposed on the image of the vascular pattern in the superficial cortex shown in (a). (d) Open-skull photograph of the rat cortical surface. B, bregma; L, lambda; M, midline; A, activated regions corresponding to whisker stimulation (4 mm × 4 mm). (e) Histology of normal lamina IV cortical barrels, located in regions A, representing the large mystacial vibrissae of the rat somatosensory system (40× magnification). Figure adapted from Ref. 3.

**Fig. 2:**
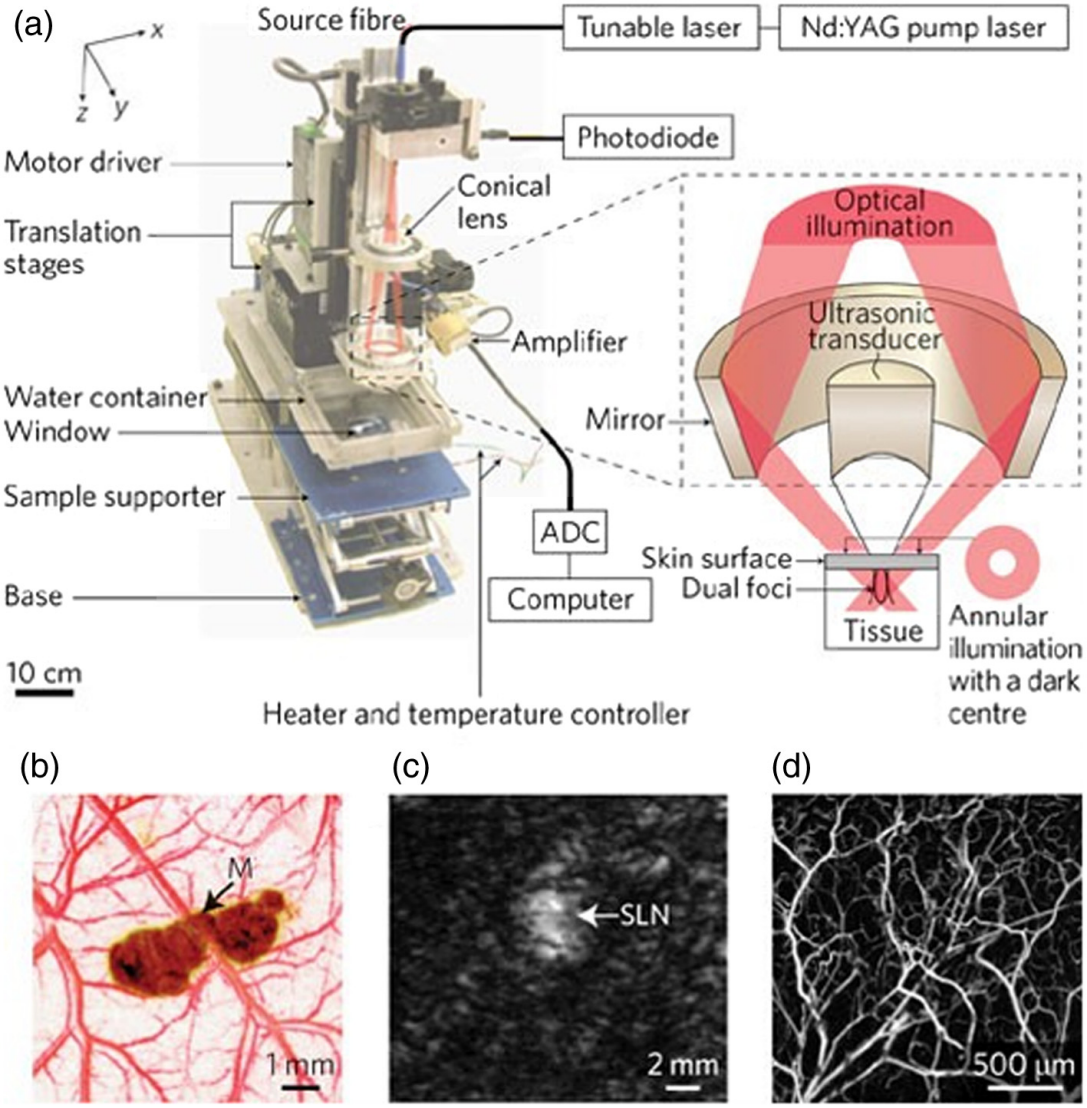
**Multiscale photoacoustic microscopy/macroscopy (PAM).** (a) Schematic of the acoustic-resolution PAM. (b) Image of a melanoma and blood vessels, acquired with a 50-MHz acoustic-resolution PAM at an axial resolution of 15  μm and a penetration limit of 3 mm. (c) Image of a sentinel lymph node (SLN) 18 mm below the laser-illumination surface, acquired with a 5-MHz acoustic-resolution PAM at an axial resolution of 144  μm and a penetration limit of 30 mm. (d) Image of the vasculature (including capillaries), acquired with an optical-resolution PAM at a lateral resolution of 5  μm and a penetration limit of 0.7 mm. Figure adapted from Ref. 8.

Prof. Wang is unquestionably one of the world’s most influential researchers in the field of biomedical photoacoustics, and has pioneered its development for structural, functional, metabolic, and molecular imaging across spatiotemporal scales.^1^^,^^2^ His contributions to photoacoustic imaging have been groundbreaking and have significantly advanced the resolution, imaging capabilities, and applications of this emerging technology. He has made seminal contributions to photoacoustic computed tomography (PACT), which combines the molecular contrast of light and the superb penetration of ultrasound for deep-tissue imaging. Notably, the landmark paper from his group in 2003 demonstrated, for the first time, noninvasive, high-resolution, functional PACT of the rodent brain^3^ (Fig. 1)—igniting an exponential growth of this field over the past two decades. Expanding upon this achievement, his group further advanced PACT with the use of optically absorbing reporter genes and photo-switchable proteins, enabling high-resolution, deep-tissue imaging of molecular probes^4^ and gene expression.^5^ Moreover, his recent developments of high-speed PACT enabled dynamic imaging of physiological changes in the human brain and breast, paving the way for clinical translation.^6^^,^^7^ In addition to his contributions to PACT, Prof. Wang has made landmark contributions to photoacoustic microscopy (PAM) across multiple spatial scales. His invention of acoustic-resolution PAM overcame the long-standing challenge of optical diffusion in light microscopy (Fig. 2).^8^ His invention of optical-resolution PAM introduced a new contrast to intravital light microscopy, complementing established technologies such as optical coherence tomography and multiphoton fluorescence microscopy.^9^ His persistent development of PAM over the years has led to a significant expansion of its spatiotemporal coverage and imaging contrast, including super-resolution PAM with nanometer resolution,^10^ high-speed PAM with 100-kHz B-scan rate,^11^ ultraviolet PAM for label-free intraoperative histology,^12^ and ultraviolet-localized mid-infrared PAM for high-resolution chemical imaging,^13^ just to name a few. Through his vision and innovative approaches, Prof. Wang has shaped the field of biomedical photoacoustics and opened numerous new possibilities for both basic research and clinical translation.

## 2 Monte Carlo Modeling of Photon Transport in Biological Tissues

Prof. Wang is one of the key contributors who established the foundation for the Monte Carlo simulation of photon transport in tissue, which has been broadly adopted by researchers worldwide in various areas of biomedical optics. Following the initial work by Prof. Jacques and others,^14^ Wang and Jacques published their seminal work on Monte Carlo modeling of light transport in multi-layers tissue (MCML)^15^ using ANSI C. They made their source code publicly available with a 173-page manual for the code.^14^ Similar to how modern Python intrinsically breaks the barriers among operational systems to ensure cross-platform compatibility, the ANSI C compiler was available in nearly all major operational systems in the 1990s, and the MCML code ensured broad applicability. Also, in MCML, Wang and Jacques carefully optimized the computational efficiency by applying several statistically sound simplifications, such as binary boundary conditions for photon packets hitting a tissue interface. As a result, MCML became the most-cited paper on Monte Carlo modeling in biomedical optics.

Since his initial contributions to the Monte Carlo modeling method itself, Prof. Wang and colleagues have applied Monte Carlo modeling to optical coherence tomography,^16^ propagation of polarized light,^17^^,^^18^ ultrasound-modulated optical tomography,^19^ thermoacoustic tomography,^20^ photoacoustic microscopy,^21^ and bioluminescent imaging,^22^ to name a few. In 2007, Prof. Wang and Prof. Wu published the book *Biomedical Optics: Principles and Imaging*, where they explained the mathematical background and programming details for Monte Carlo modeling, provided well-thought practice questions, and applied Monte Carlo simulations to investigate various tissue optical properties.

## 3 Compressed Ultrafast Photography

**Fig. 3:**
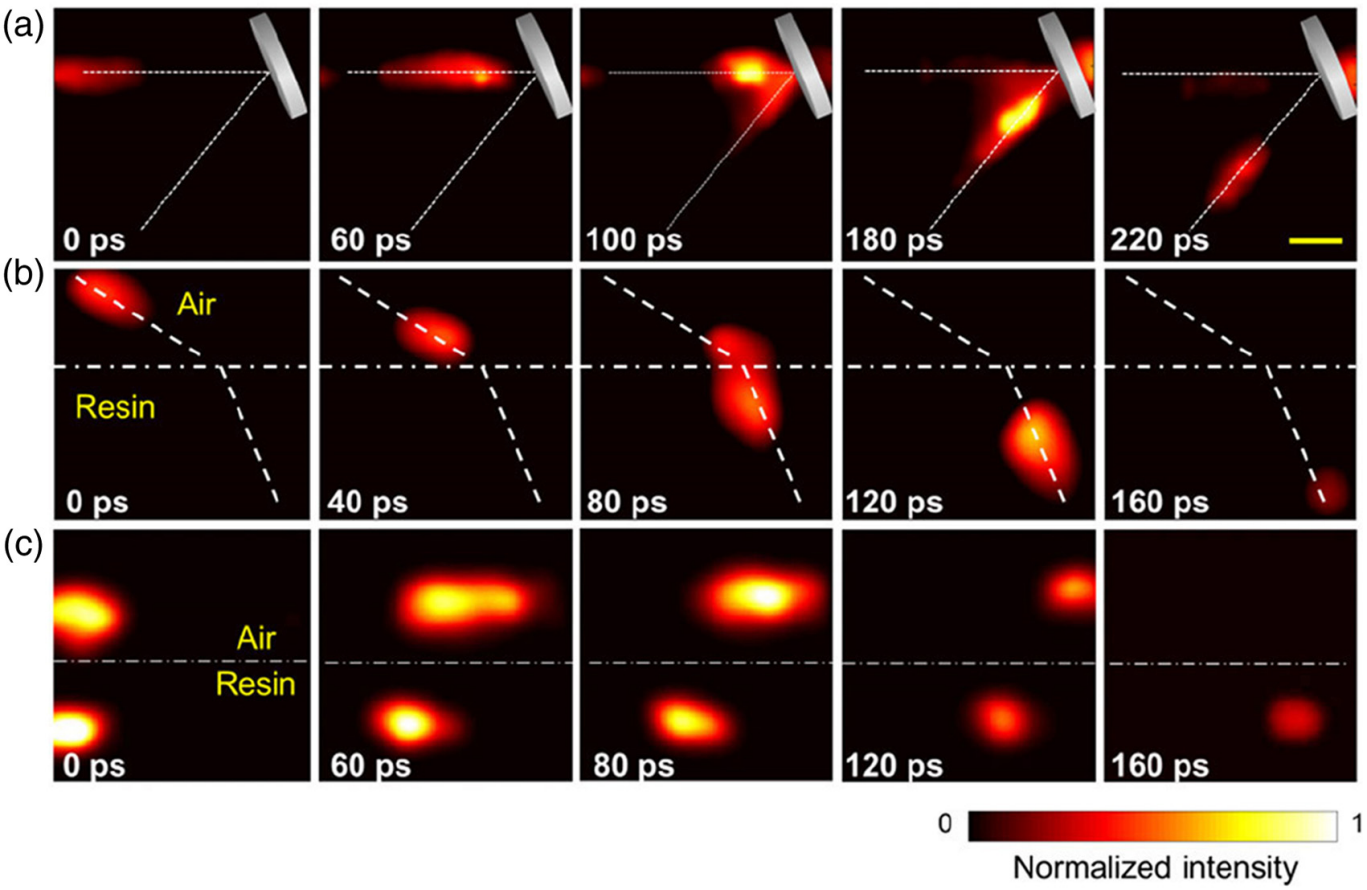
**CUP of light propagation.** (a) Laser pulse reflected from a mirror. (b) Laser pulse refracted from an air-resin interface. (c) Laser pulses racing in air and resin. Scale bar (yellow, top right image), 10 mm. Figure adapted from Ref. 23.

Ultrafast cameras stand at the forefront of both fundamental research and practical applications in the sciences. Yet, their ability to capture extensive 2D/3D scenes has long been hindered by electronic bandwidth limitations. Prof. Wang met this challenge head-on by pioneering the integration of compressed sensing into streak camera data acquisition. His revolutionary method, compressed ultrafast photography (CUP), marked a pivotal advancement in ultrafast imaging, as showcased on the cover of the journal *Nature* in 2014 (Fig. 3).^23^

CUP represents a groundbreaking technique that transcends traditional limitations, allowing for unprecedented 2D imaging of transient events at an astonishing frame rate of 219 trillion frames per second.^24^^,^^25^ What distinguishes the CUP technique from conventional approaches is its reliance on passive data acquisition, leveraging the camera’s inherent capabilities for temporal resolution rather than requiring active illumination. This unique attribute vastly broadened the scope of CUP applications, enabling imaging not only in transmitted light but also in fluorescence, bioluminescence, and chemiluminescence. The ingenuity of the CUP technique garnered significant recognition; it has been recognized as the state-of-the-art ultrafast imaging method applicable to a wide array of existing fluorescence tools. CUP serves as a universal imaging platform adaptable to various optical instruments, including microscopes and telescopes, elevating their imaging speed to unprecedented levels and catalyzing new scientific breakthroughs across scales, from cellular organelles to distant galaxies. The versatility of CUP has been demonstrated across numerous scientific domains, including fluorescence lifetime imaging microscopy,^26^^,^^27^ visualization of stochastic physical events,^28^^,^^29^ and imaging through scattering media,^30^

## 4 Time Reversal and Wavefront Shaping in Scattering Media

The ability to focus light deep into biological tissue has been a holy grail for optical imaging scientists. The ability to achieve this will enable tissue imaging beyond the optical diffusion limit with true optical resolution while utilizing all the contrast mechanisms available to optical imaging. However, while there are no physical laws preventing the tight focusing of the light deep into the scattering media, technical challenges are abundant. Due to random scattering, focusing beyond the diffusion limit in the biological tissue requires estimation and generation of a very complex wavefront of light illuminating the tissue, such that constructive interference is created at the focus deep inside the tissue. Moreover, biological tissue is a highly dynamic scattering medium, leading to a rapid change of the optical speckle pattern both inside and outside the tissue. The electric field autocorrelation functions measured by technologies relying on dynamic light scattering in human tissue, such as diffused correlation spectroscopy, typically decay in much less than a millisecond. This leaves little time to measure the optimal wavefront shape and apply it to focus the light deep into the tissue.

**Fig. 4:**
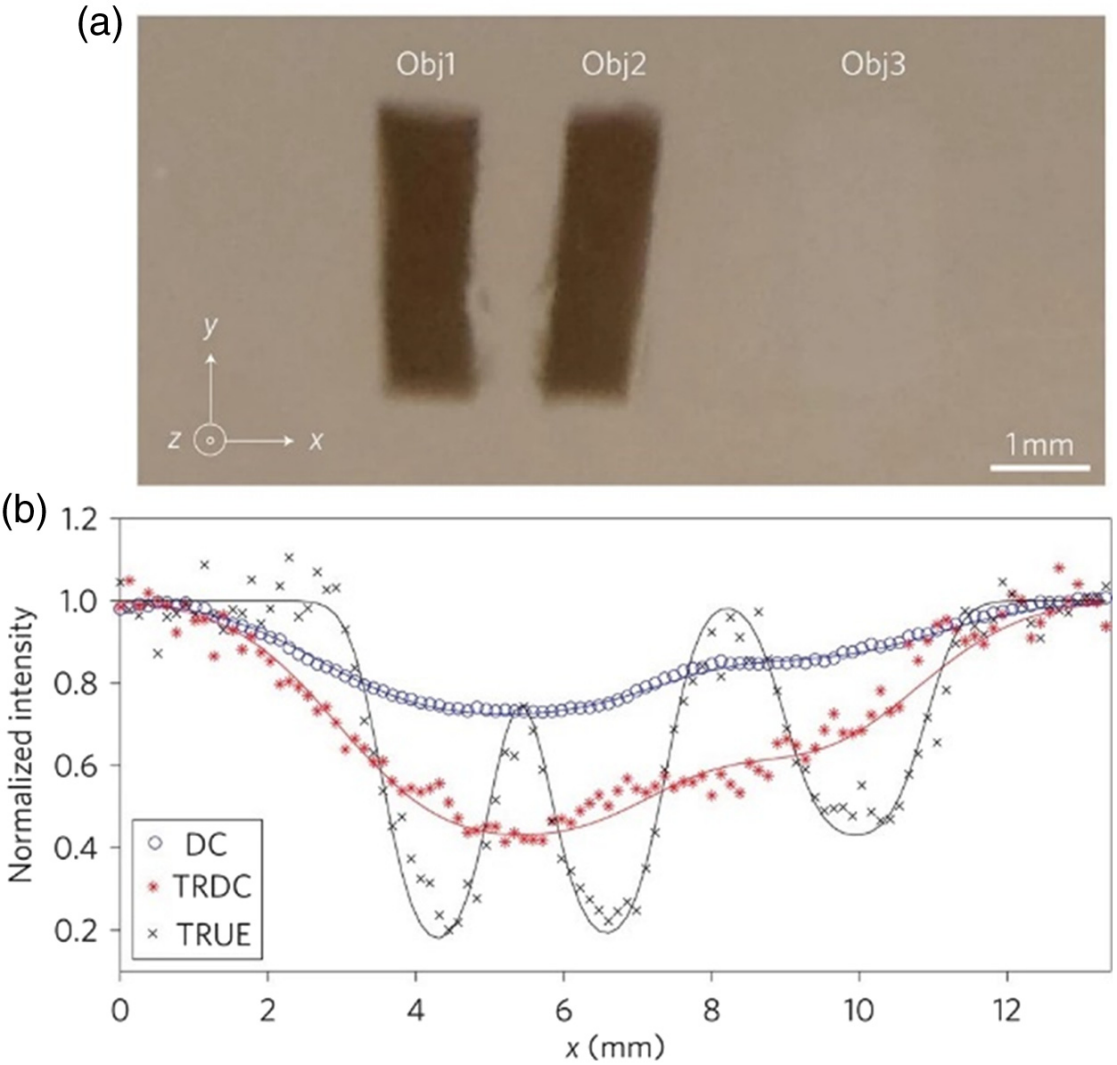
**Demonstration of a time-reversed ultrasonically encoded (TRUE) optical focusing in a scattering phantom.** (a) Photograph of the imaged sample dissected at the middle plane containing two absorbing objects (Obj1 and Obj2) and one scattering object (Obj3). The object dimensions were x=1.3  mm, y=4.5  mm and z=1  mm for the two absorbing objects and x=1.7  mm, y=4.5  mm and z=0.6  mm for the scattering object. The full dimensions of the sample were x=y=60  mm and z=10  mm. (b) Comparison of the normalized direct current (DC), time-reversed direct current (TRDC) and TRUE images of the sample. The absolute strengths of the TRDC and TRUE signals were ∼3,000  mV and ∼30  mV, respectively. The objects could not be distinguished in the DC and TRDC images, but in the TRUE image the objects were clearly shown against the background with 61% contrast for the absorbing objects and 31% contrast for the scattering object. In (b), symbols represent experimental data and solid curves represent Gaussian fits. Figure adapted from Ref. 31.

To address these challenges, in 2011, Prof. Wang’s team proposed a new fast wavefront-shaping technique based on ultrasound tagging of light as a guide star and using the photorefractive crystal to quickly time-reverse the field and focus the light back into the tissue (Fig. 4).^31^ This technique, referred to as time-reversed ultrasonically encoded (TRUE) optical focusing,^31^ opened an innovative new research direction in the field of wavefront shaping that addressed the troubling challenge of very fast speckle decorrelation times in biological tissues. During the subsequent decade, a number of high-impact papers were published by Prof. Wang’s group that both significantly improved the TRUE method and established other novel approaches (e.g., TRAP^32^) for fast wavefront shaping. Thanks to Prof. Wang’s work that reshaped the wavefront-shaping research filed,^33^^–^^36^ we are today closer than ever before to witnessing the first practical applications of light focusing beyond the optical diffusion limit in thick biological tissues.

In summary, it is truly an honor to celebrate Prof. Wang’s contributions via this special issue of JBO. His passion for biomedical optics research has impacted generations of students, post-doctoral researchers, and colleagues alike. We are humbled and grateful to call him a mentor, colleague, and friend, and hope you will enjoy the special issue.
